# Photo-activation of Single Molecule Magnet Behavior in a Manganese-based Complex

**DOI:** 10.1038/srep23785

**Published:** 2016-03-30

**Authors:** Ahmed Fetoh, Goulven Cosquer, Masakazu Morimoto, Masahiro Irie, Ola El-Gammal, Gaber Abu El-Reash, Brian K. Breedlove, Masahiro Yamashita

**Affiliations:** 1 Department of Chemistry, Graduate School of Science, Tohoku University, 6-3 Aramaki-Aza-Aoba, Aoba-ku, Sendai 980-8578, Japan; 2 Department of Chemistry, Faculty of Science, Mansoura University, Mansoura, Egypt; 3 Core Research for Evolutional Science and Technology (CREST), Japan Science and Technology (JST), 4-1-8 Kawaguchi, Saitama 332-0012, Japan; 4 Department of Chemistry and Research Center for Smart Molecules, Rikkyo University, Nishi-Ikebukuro 3-34-1, Toshima-ku, Tokyo, Japan

## Abstract

A major roadblock to fully realizing molecular electronic devices is the ability to control the properties of each molecule in the device. Herein we report the control of the magnetic properties of single-molecule magnets (SMMs), which can be used in memory devices, by using a photo-isomerizable diarthylenthene ligand. Photo-isomerization of the diarylethene ligand bridging two manganese salen complexes with visible light caused a significant change in the SMM behavior due to opening of the six-membered ring of diarylethene ligand, accompanied by reorganization of the entire molecule. The ring-opening activated the frequency-dependent magnetization of the complex. Our results are a major step towards the realization of molecular memory devices composed of SMMs because the SMM behaviour can be turned on and off simply by irradiating the molecule.

Single-molecule magnets (SMMs) have the ability to keep their magnetization without external field and, thus, can be used for information storage at the molecular level or in spintronic devices^1^. In order to use SMMs in information storage devices, the magnetic properties of the SMMs must show hysteresis, and it must be possible to control them by applying external stimuli, such as light, electric, pressure, *etc.*, which can be accomplished by including multifunctional ligands^2^,^3^,^4^,^5^. Photo-switchable magnetic materials have become quite important in the field of high-density information storage media^6^,^7^,^8^. On the other hand, the slow relaxation, due to its short lifetime, can be used to design dynamic random access memory (DRAM) device and to investigate spintronics and quantum computing. We have incorporated photoactive diarylethene derivatives with two hydroxo groups (dae), which reversibly isomerizes between open (dae-o) and closed forms (dae-c) when irradiated with UV or visible light (Figure S1), respectively, into SMM complexes to control the magnetic properties of the SMMs^9^,^10^,^11^.

Recently, we have reported that the dae ligand in one-dimensional (1D) assemblies of Mn_4_ SMMs with *S* = 9 can be photo-isomerized to afford [Mn_4_(hmp)_6_(dae-o)_2_(ClO_4_)_2_]·6H_2_O (Mn_4_-o) and [Mn_4_(hmp)_6_(dae-c)_2_(H_2_O)_2_](ClO_4_)_2_·CH_3_CN·4H_2_O (Mn_4_-c) (Hhmp: 2-hdroxymethylpyridine)^12^. The photocyclization of Mn_4_-o by UV irradiation does not affect the magnetic properties. However, in the case of Mn_4_-c, the photocycloreversion by irradiation with visible light changed the inter Mn_4_ arrangement between the neighboring 1D chains, thus enhancing the interchain Mn_4_ interactions. In addition, two photo-switchable assemblies of 3*d*4*f* cores, [Cu^II^Tb^III^(L)(NO_3_)_3_] (H_2_L: 1,3-bis((3-methoxysalicylidene)amino) propane), containing dae have been reported^13^. Similar to Mn_4_, irradiation of the closed form with visible light induces a change in the magnetic properties due to a change in the intermolecular interactions. For 2D coordination-layer topologies, in which carboxylato bridged Ln_2_ units are linked together by dae^2−^ anions into grid-like frameworks^14^,^15^, the relaxation behavior in the quantum-tunneling regime is affected by the photoisomerization of dae. Thus, photochromic molecules are useful for tuning the magnetic behavior of complexes without switching on/off the slow magnetic relaxation.

In this study, the SMM behavior of a complex comprised of [Mn_2_(salen)_2_(H_2_O)_2_](ClO_4_)_2_ SMM units bridged by dae-c (**1c**) could be turned on by irradiating it with visible light, which affords a complex containing dae-o (**1c-Vis**). Crystal structure analyses of **1c** and **1c-Vis** showed that dae-c could be converted to dae-o in the solid state. In addition, UV-vis spectroscopy was used to confirm that the photo-conversion was reversible and repeatable. From static and dynamic magnetic measurements, only the complex with dae-o exhibited SMM behavior. This is unlike our previous complexes, where the magnetic behaviour is only modified due to opening/closing of the dae ligand. In other words, for the first time, we show that photo-isomermization of the dae ligand can be used to turn on and off the SMM behaviour.

## Results and Discussion

### Structural Description

**Figure 1:**
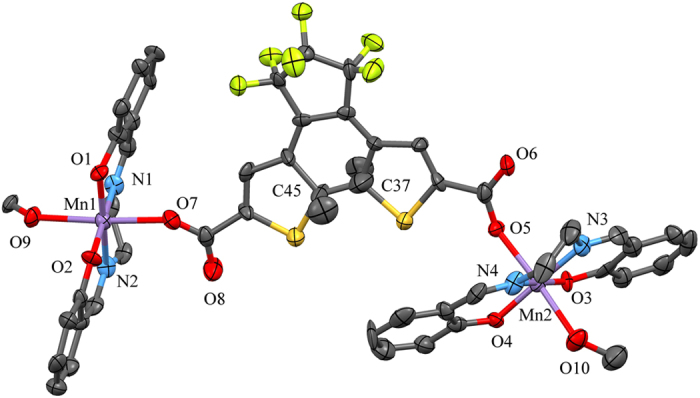
ORTEP diagrams of **1c**. Representation with 30% thermal ellipsoid. Solvent molecules and hydrogen atoms were omitted for clarity.

Crystallographic data for **1c** and **1c-Vis** are summarized in Table S1. **1c** crystallized in the orthorhombic space group *P*2_1_/*cn* (Fig. 1). The Mn^III^ ions are hexacoordinated with an N_2_O_2_ atom set from 2,2′-ethylenebis(nitrilomethylidene)phenol (salen^2−^), one oxygen atom from the carboxylato group of dae-c^2−^, and one oxygen atom from a coordinated methanol molecule. The two Mn^III^ ions exhibited Jahn-Teller distortion with elongation of the oxygen-metal distance perpendicular to the N_2_O_2_ atom set of salen^2−^ (O9-O7 and O5-O10 axes for Mn1 and Mn2, respectively) (Table S2).

The dae-c^2−^ ligand acts as a monodentate ligand bridging Mn(salen) monomers via the carboxylato groups. In the packing diagrams of **1c** (Figure S2), Mn(salen) and dae-c^2−^ units are not organized in organic and inorganic sub networks. Two non-coordinated methanol solvent molecules are present in the asymmetric unit. No π-π interactions were observed in the crystal. However, hydrogen bonds between the non-coordinated and coordinated oxygen atoms of the dae-c^2−^ ligand and non-coordinated methanol molecules were found. In addition, there are hydrogen bonds between the fluorine atoms of dae-c^2–^ and the hydrogen atoms of the solvent molecules. The intramolecular Mn^III^**···**Mn^III^ distance across the dae-c^2−^ ligand was determined to be 13.943 Å, whereas the nearest intermolecular distance was found to be 6.533 Å.

Crystals of **1c-Vis** were obtained by irradiating those of **1c** with sunlight, and it crystallized in the *P*2_1_/*cn* space group. The coordination modes of the Mn^III^ ions are similar to those in **1c** (Figure S3) (Table S2). Since photo-isomerization of the complex induced constraint in the crystal, the atoms could not be refined anisotropically. Packing diagrams for **1c-Vis** (Figure S4) are similar to those for **1c.** The intramolecular Mn^III^**···**Mn^III^ distance across the dae-o^2−^ ligand was determined to be 15.147 Å, and the nearest interunit Mn^III^**···**Mn^III^ distance was found to be 8.168 Å. These distances are larger than those in **1c**

During photo-opening of the dae ligand, the non-coordinated methanol evaporated. The solvent available void by unit cell was determined to be 277.6 Å^3^ in **1c-Vis**, whereas it was determined to be 479.1 Å^3^ in **1c**. In the case of the coordinated methanol molecules, only the oxygen atoms could be localized, and we could not determine if the carbon atoms of the methanol molecules were too disordered to be localized or if the methanol molecules were actually water molecules.

The crystal structure of **1c-Vis** after UV irradiation could not be obtained due to a loss of crystallinity.

### UV-Visible Spectroscopy

Since the dae ligand undergoes reversible isomerization between closed and opened ring forms upon visible light (opening process) and UV irradiation (closing process) in solution and the solid state^16^,^17^,^18^,^19^,^20^,^21^,^22^, we studied the photo-isomerization processes in the solid state using KBr pellets of **1c** before and after visible irradiation (Figure S5). In the absorption spectra, there were two bands around 380 and 580 nm, which were assigned to be π–π* transitions of the dae^2−^ ligand^23^. Irradiation of the KBr pellets of **1c** with visible light (*λ* > 480 nm) caused a pronounced color change from black to pale brown, which is characteristic of dae^2−^-c and dae^2–^-o, respectively. After visible light irradiation, the two absorption bands disappeared due to ring opening. After UV light irradiation (*λ* > 365 nm) of **1c-Vis**, the initial spectrum was recovered, meaning that the photocyclization reaction was reversible. In other words, isomerization between the closed and opened isomers occurs reversibly in the solid state.

### Static Magnetic Properties

**Figure 2:**
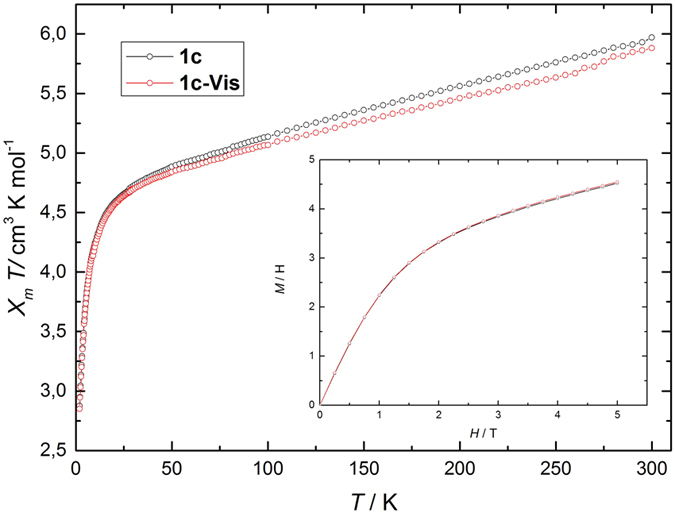
Comparison of the static magnetic properties of **1c** and **1c-Vis**. Temperature dependence of *χ*_m_*T*. Insets: *M vs. H* at 1.82 K.

The temperature dependence of the magnetic susceptibilities (*χ*_m_*T*) of polycrystalline samples of **1c** and **1c-Vis** were measured in the *T* range of 2–300 K and are shown in Fig. 2. *χ*_m_*T* values at room *T* were determined to be 5.97 and 5.88 cm^3^ mol^−1^ K for **1c** and **1c-Vis**, respectively. These values are comparable with the expected value of 6.0 cm^3^ mol^−1^ K for two non-interacting high-spin Mn^III^ ions (*S* = 2). The *χ*_m_*T* values decreased monotonically from 300 to 30 K and sharply between 30 K and 2 K, indicating the presence of dipole-dipole antiferromagnetic interactions.

The magnetic data obeys the Curie-Weiss law, affording Curie constant *C* = 5.81 and 5.71 cm^3^ K mol^−1^ and Weiss temperature *θ* = −1.16 and −1.12 K for **1c** and **1C-Vis**, respectively. The *C* values obtained from fitting are close to the spin-only value of 6.11 cm^3^ K mol^−1^ corresponding to two high-spin Mn^III^ ions. Moreover, the negative *θ* values confirm that there are antiferromagnetic interactions between the Mn^III^ ions.

The field dependence of the magnetization, *M vs.* applied field (*H*), at 1.82 K for **1c** and **1c-Vis** were similar. *M* of the complexes increased continually with an increase in *H* up to 4.55 N*μ*_B_ at 5 T with a decrease in the slope at 1.5 T. The *M* values did not saturate up to 8.00 N*μ*_B_ (*S* = 4), which is the *M* value expected for two high-spin Mn^III^ ions. The absence of saturation and the observed slowdown were ascribed to the antiferromagnetic interactions and magnetic anisotropy due to the Jahn-Teller distortion of the high-spin Mn^III^ ions. Hysteresis was not observed for either complex (Figure S6).

### Dynamic Magnetic Properties

**Figure 3:**
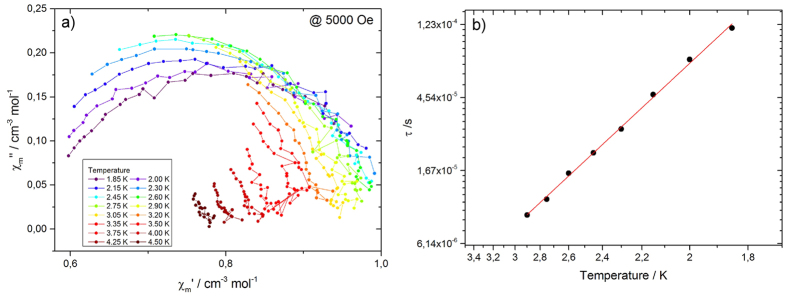
Slow magnetic relaxation of **1c-Vis**. Left: Cole-Cole plots at several *T* in an *H* of 5000 Oe. Right: Arrhenius plots.

Ac magnetic susceptibilities were obtained for polycrystalline samples of **1c** and **1c-Vis** as a function of *T, H* and frequency. No out-of-phase signal was observed for **1c,** and weak frequency dependence was observed for **1c-Vis** (Figure S7). In order to suppress potential quantum tunneling relaxation of the *M*, an *H* was applied to cause a mismatch in the +*m*_*s*_ and −*m*_*s*_ magnetization levels^24^. For **1c**, an *H* less than 4000 Oe was not sufficient to observe a peak in the out-of-phase signal, characteristic of slow relaxation of the magnetization (Figure S8). Detailed *H*-dependent ac measurements at 1.9 K showed that an *H* of 5000 Oe was needed to observe slow magnetic relaxation for **1c-Vis** (Figure S9). In-phase (*χ*′) and out-of-phase magnetic susceptibilities (*χ*″) for **1c-Vis** were determined in an *H* of 5000 Oe at various *T* (Fig. 3a). The relaxation time *τ* was determined by using a generalized Debye model for a distributed single relaxation process of *M*, as show in equations (1) and (2), where *χ*_adia_ and *χ*_iso_ are the adiabatic and isotherm susceptibilities, respectively, *τ* is the relaxation time, and *α* the distribution coefficient of *τ*^25^,^26^.


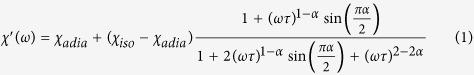



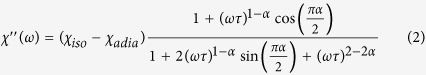



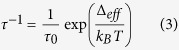


The obtained *τ* values for **1c-Vis** at several *T* were fitted using the Arrhenius law (equation 3), where *τ*_0_ is the pre-exponential factor and Δ_*eff*_ is the effective energy barrier for the reversal of *M* (Fig. 3b). Δ_*eff*_/*k*_B_ and *τ*_0_ were determined to be 13.13 K and 1.008 × 10^−7^ s at 5000 Oe, respectively. These values are comparable to those determined for other [Mn_2_] SMMs^27^,^28^.

The magnetic properties significantly changed upon photo-isomerization of the ligand. The closed form did not exhibit slow magnetic relaxation, whereas the open form exhibited clear frequency-dependent magnetic relaxation. The change in the magnetic properties is due to two reasons. First, the slight change in inter- and intra-unit Mn^III^**···**Mn^III^ distances upon photo-isomerization of the ligand could affect the magnetic interactions between the magnetic centers, although this is not supported by the results of the dc measurements. Second, the *π*-conjugation of the two thiophene rings are delocalized over the entire molecule in the closed ring form, whereas it is localized in the open ring form. The change in *π*-conjugation of the dae moiety upon irradiation can change the super-exchange interactions between the two Mn^III^ ions. Due to the extended conjugation *π-*system, the quantum tunneling relaxation of *M* is dominant in the closed ring form. To clearly determine the mechanism and origin of the photo control of the magnetic properties, theoretical calculations, including the aromaticity of the ligand, the intramolecular interactions trough the *π*-conjugated bond and through dipole-dipole interactions, the change of the coordination geometry around the Mn ions, and the intermolecular interactions, are needed. Due to the scope of this report, the results of the computational studies will be reported later.

**Figure 4:**
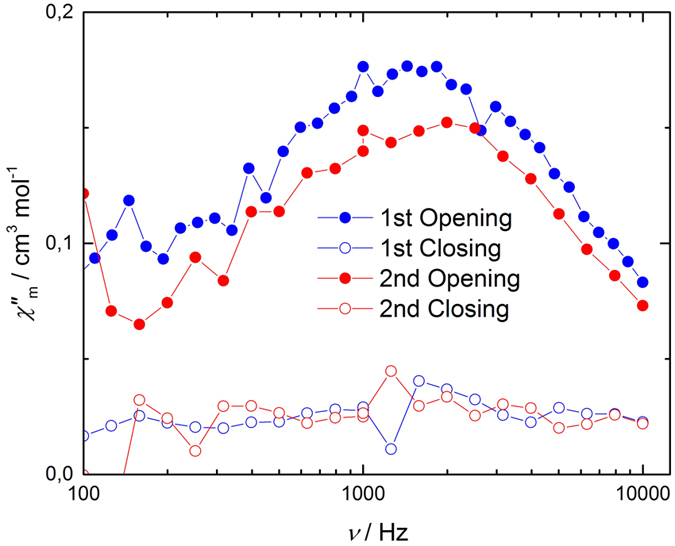
Switching on/off of the SMM behavior. Frequency dependence of out-of-phase susceptibility for the complex after opening and closing process at 1.9 K.

To determine the reversibility and repeatability of this activation, the sample was successively irradiated with visible and UV light over one day at room temperature, and magnetic measurements were performed at 1.9 K at each wavelength (Fig. 4). After visible irradiation, a clear peak appeared, whereas no peak appeared after irradiation with UV light, demonstrating that the SMM behavior could be switched on/off reversibly and repeatedly. The peak intensity after the second opening decreased slightly in comparison to the first one due to the fatigue of the complex, where the dae ligand itself or its coordination to Mn ion gradually degraded.

## Conclusions

Irradiation of a photochromic dae ligand was used to repeatedly turn on and off the SMM behaviour of Mn(salen) complexes. When the complex with the closed ring form of dae (**1c**) was converted to **1c-Vis** by irradiating with visible light, slow relaxation of the magnetization was observed, whereas it was not for **1c**. This change in behaviour is due to slight changes in inter- or intra-unit Mn^III^**···**Mn^III^ distances upon photoisomerization of dae. In addition, the superexchange interactions between the two Mn^III^ ions caused by the *π*-conjugation of the two thiophene rings affects the magnetic properties of the two forms. To the best of our knowledge, this is the first example of a coordination assembly where the magnetic properties are activated upon irradiation with visible light, and it is a significant advance in the area of molecular memory using SMMs.

## Methods

### General Procedures and Materials

All chemicals and solvents were purchased from Tokyo Chemical Industry Co. Ltd. or Wako Pure Chemical Industries Ltd. and used as received. The diarylethene ligand H_2_dae-c and [Mn_2_(salen)_2_(H_2_O)_2_](ClO_4_)_2_ were synthesized following reported procedures^29^,^30^. Synthesis and characterization were carried out in the dark to prevent the closed ring isomer from undergoing photo-cycloreversion.

### Synthesis of [{Mn(salen)MeOH}_2_(dae-c)]·(MeOH)_2_ (1c)

To a solution of [Mn_2_(salen)_2_(H_2_O)_2_](ClO_4_)_2_ (69.87 mg, 0.08 mmol) in 10 ml of methanol, a solution of H_2_dae-c (18.23 mg, 0.04 mmol) in 5 ml of methanol was added. The resulting solution was stirred for 15 min at 50 °C and then filtered. Black crystals of **1c** were obtained by slow diffusion of acetonitrile into the reaction solution over 1 week. The crystals were collected by filtration. Anal. Calcd (%) for C_53_H_52_N_4_F_6_Mn_2_O_12_S_2_: C 51.96; H 4.28; N 4.57. Found (%): C 51.80; H 4.18; N 4.63.

### Physical Measurements

Solid-state UV/Vis absorption spectra of **1c** were measured using a KBr matrix on a Shimadzu UV-3100 spectrophotometer before and after irradiation with visible light of *λ* = 480 nm (**1c** and **1c-Vis** respectively) and after irradiation at *λ* = 356 nm (**1c-UV**). Measurements were carried out at room temperature.

Dc susceptibility measurements were performed on a Quantum Design MPMS-5S superconducting quantum interference device (SQUID) magnetometer with a polycrystalline sample in applied magnetic fields of 100 Oe in the range of 1.8–20 K, 500 Oe in the range of 18–50 K, and 1000 Oe in the *T* range of 45–300 K. Experimental data were corrected for the sample holder contribution, and the diamagnetism of the sample was calculated from Pascal’s constants. The ac susceptibilities was acquired on a Quantum Design PPMS-6000 physical property measurement system with an ac field amplitudes of 3 Oe below 1000 Hz and 1 Oe over 1000 Hz with and without a static dc field. After measuring the magnetic properties of **1c**, the samples were alternately irradiated with sunlight and UV light for 1 day outside of the measurement system at room temperature, and the magnetic properties were measured at 1.9 K after each irradiation.

Single crystals were mounted on a glass rod, and crystallographic data were collected on a Rigaku Saturn70 CCD diffractometer with graphite-monochromated Mo *Kα* radiation (*λ* = 0.71073 nm) produced by a VariMax microfocus X-Ray rotating anode source at 103 K. Data processing was performed using the Crystal Clear crystallographic software package. The structures were solved by using direct methods via SIR-92 or SIR-2011^31^ and refined using the full-matrix least-squares technique included in SHELXL-2013^32^. The final cycles of full-matrix least-squares refinements on F^2^ converged with unweighted and weighted agreement factors of *R*_1_ = Σ||*F*_o_|−|*F*_c_||/Σ|*F*_o_| (*I* > 2.00*σ*(*I*) for *R*_1_), and *wR*_2_ = [Σw(*F*_o_^2^ − *F*_c_^2^)^2^]/Σw(*F*_o_^2^)^2^]^1/2^ (all reflections), respectively. Anisotropic thermal parameters were assigned to all non-hydrogen atoms for **1c**. The hydrogen atoms were set in calculated positions and refined using a riding model with a common fixed isotropic thermal parameter. CCDC-1443151 and 1443152 contains the supplementary crystallographic data for this paper. These data can be obtained free of charge from The Cambridge Crystallographic Data Centre via www.ccdc.cam.ac.uk/data_request/cif.

## Additional Information

**How to cite this article**: Fetoh, A. *et al*. Photo-activation of Single Molecule Magnet Behavior in a Manganese-based Complex. *Sci. Rep.* **6**, 23785; doi: 10.1038/srep23785 (2016).

## Supplementary Material

Supplementary Information

Supplementary Information
