# Supplementary material for: Photo-activation of Single Molecule Magnet Behavior in a Manganese-based Complex
Source: Sci Rep. 2016 Mar 30;6:23785. doi: 10.1038/srep23785 (PMC4824451; doi:10.1038/srep23785)
Supplement: Supplementary Information [file srep23785-s1.pdf]

Photo-activation of Single Molecule Magnet Behavior in a Manganese-based Complex.

Ahmed Fetoh,[a,b] Goulven Cosquer,*[,a,c] Masakazu Morimoto,[d] Masahiro Irie,[d] Ola El-Gammal,[b] Gaber Abu El-Reash,[b] Brian K. Breedlove,[a,c] and Masahiro Yamashita*[,a,c]

SUPPLEMENTARY INFORMATION

Experimental Section

General Procedures and Materials. All chemicals and solvents were purchased from Tokyo Chemical Industry Co. Ltd. or Wako Pure Chemical Industries Ltd. and used as received. The diarylethene ligand H_2dae-c and $[Mn_2(salen)_2(H_2O)_2](ClO_4)_2$ were synthesized following reported procedures.[29,30] Synthesis and characterization were carried out in the dark to prevent the closed ring isomer from undergoing photo-cycloreversion.

Synthesis of $[\{Mn(salen)MeOH\}_2(dae\text{-}c)]\cdot(MeOH)_2$ (1c). To a solution of $[Mn_2(salen)_2(H_2O)_2](ClO_4)_2$ (69.87 mg, 0.08 mmol) in 10 ml of methanol, a solution of H_2dae-c (18.23 mg, 0.04 mmol) in 5 ml of methanol was added. The resulting solution was stirred for 15 min at 50 °C and then filtered. Black crystals of **1c** were obtained by slow diffusion of acetonitrile into the reaction solution over 1 week. The crystals were collected by filtration. Anal. Calcd (%) for $C_{53}H_{52}N_4F_6Mn_2O_{12}S_2$: C 51.96; H 4.28; N 4.57. Found (%): C 51.80; H 4.18; N 4.63.

Physical Measurements. Solid-state UV/Vis absorption spectra of **1c** were measured using a KBr matrix on a Shimadzu UV-3100 spectrophotometer before and after irradiation with visible light of λ = 480 nm (**1c** and **1c-Vis** respectively) and after irradiation at λ = 356 nm (**1c-UV**). Measurements were carried out at room temperature.

Dc susceptibility measurements were performed on a Quantum Design MPMS-5S superconducting quantum interference device (SQUID) magnetometer with a polycrystalline sample in applied magnetic fields of 100 Oe in the range of 1.8–20 K, 500 Oe in the range of 18–50 K, and 1000 Oe in the T range of 45–300 K. Experimental data were

corrected for the sample holder contribution, and the diamagnetism of the sample was calculated from Pascal's constants. The ac susceptibilities was acquired on a Quantum Design PPMS-6000 physical property measurement system with an ac field amplitudes of 3 Oe below 1000 Hz and 1 Oe over 1000 Hz with and without a static dc field. After measuring the magnetic properties of **1c**, the samples were alternately irradiated with sunlight and UV light for 1 day outside of the measurement system at room temperature, and the magnetic properties were measured at 1.9 K after each irradiation.

Single crystals were mounted on a glass rod, and crystallographic data were collected on a Rigaku Saturn70 CCD diffractometer with graphite-monochromated Mo $K\alpha$ radiation (λ = 0.71073 nm) produced by a VariMax microfocus X-Ray rotating anode source at 103 K. Data processing was performed using the Crystal Clear crystallographic software package. The structures were solved by using direct methods via SIR-92 or SIR-2011[31] and refined using the full-matrix least-squares technique included in SHELXL-2013.[32] The final cycles of full-matrix least-squares refinements on F^2 converged with unweighted and weighted agreement factors of $R_1 = \Sigma||F_o|-|F_c||/\Sigma|F_o|$ ($I > 2.00\sigma(I)$ for R_1), and $wR_2 = [\Sigma w(F_o^2 - F_c^2)^2]/\Sigma w(F_o^2)^2]^{1/2}$ (all reflections), respectively. Anisotropic thermal parameters were assigned to all non-hydrogen atoms for **1c**. The hydrogen atoms were set in calculated positions and refined using a riding model with a common fixed isotropic thermal parameter.

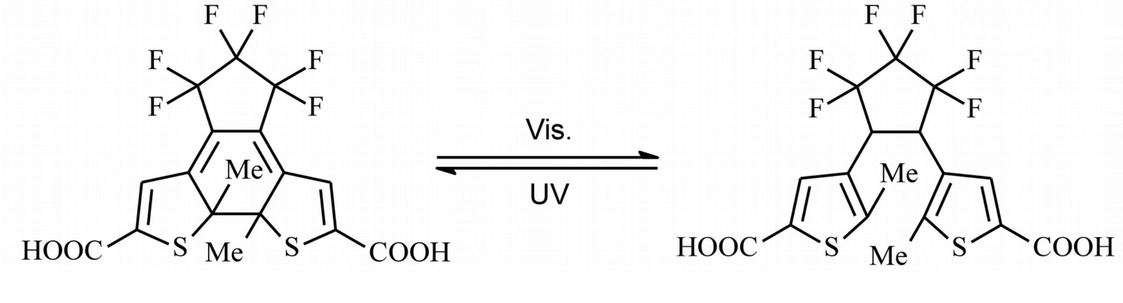

Figure S1. Photochromic dae ligand in the close and open form.

	**1c**	**1c-Vis**
Formula	$C_{53}H_{52}N_4F_6Mn_2O_{12}S_2$	$C_{51}H_{44}N_4F_6Mn_2O_{10}S_2$
Formula Weight	1125.00	1160.91
Temperature / K	103.15	103.15
Crystal System	orthorhombic	orthorhombic
Space Group	$P2_1/cn$	$P2_1/cn$
a / Å	6.5329(8)	6.6128(5)
b / Å	26.757(4)	25.0811(19)
c / Å	30.620(4)	30.7368(2)
V / Å^3	5352.4	5097.89
Z	4	4
D_{calc} / g/cm^3	1.52	1.60
F_{000}	2520	2520
μ(Mo Kα) /mm^{-1}	0.637	0.669
Data measured	18514	13217
Data unique	6905	5631
No. Variables	712	288
R_1 ($I > 2.00\sigma(I)$)	0.0745	0.3013
wR_2 (All reflections)	0.0945	0.5092
Goodness of fit	1.037	1.188

Table S1. Crystallographic data for **1c** and **1c-Vis**

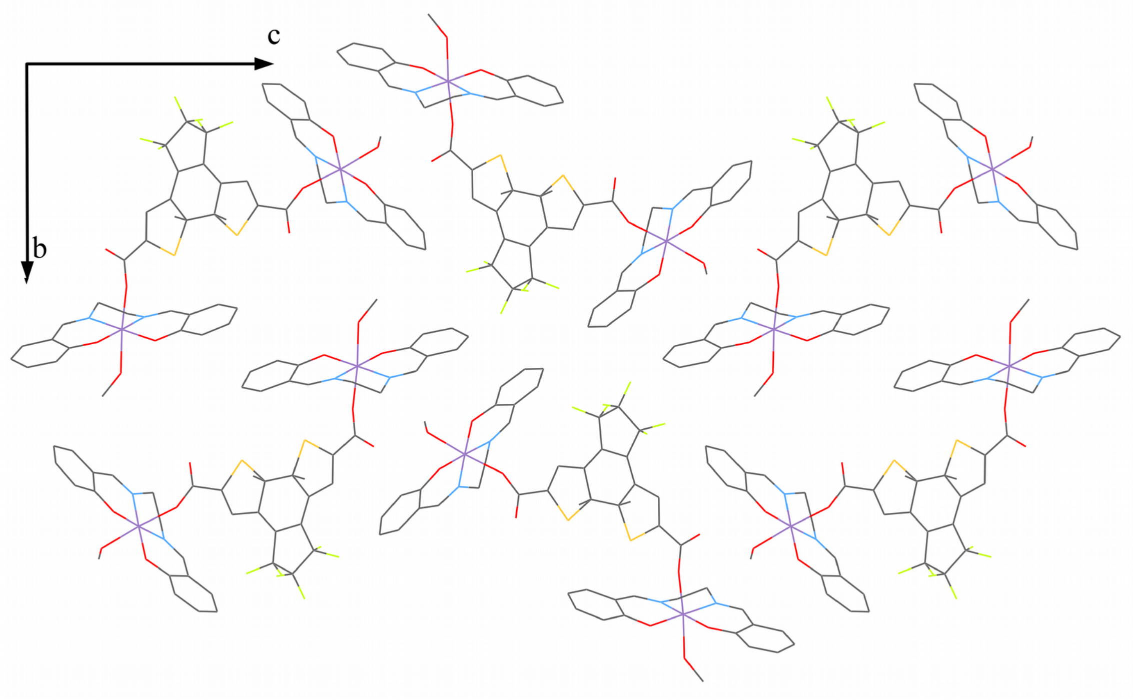

Figure S2. Packing diagrams of **1c** projected along bc plane. The hydrogen atoms and the non-coordinated methanol molecules are omitted for clarity.

	**1c**	**1c-Vis**
Mn(1)-O(7)	2.157 Å	2.001 Å
Mn(1)-O(9)	2.261 Å	2.365 Å
Mn(2)-O(5)	2.180 Å	2.177 Å
Mn(2)-O(10)	2.284 Å	2.153 Å
O(7)-Mn(1)- O(9)	175.20°	169.99°
O(5)-Mn(2)- O(10)	174.00°	169.36°

Table S2. Selected bond lengths and angles for **1c** and **1c-Vis.**

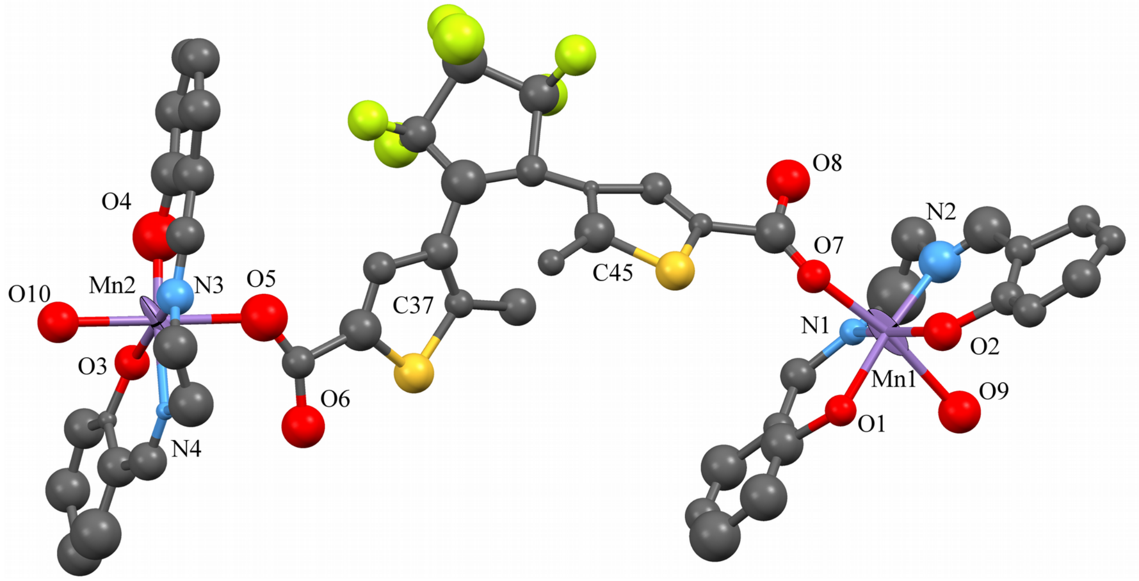

Figure S3. ORTEP view of **1c-Vis** measured at 103.15 K, with 30% thermal ellipsoid. Solvent molecules and hydrogen atoms were omitted for clarity.

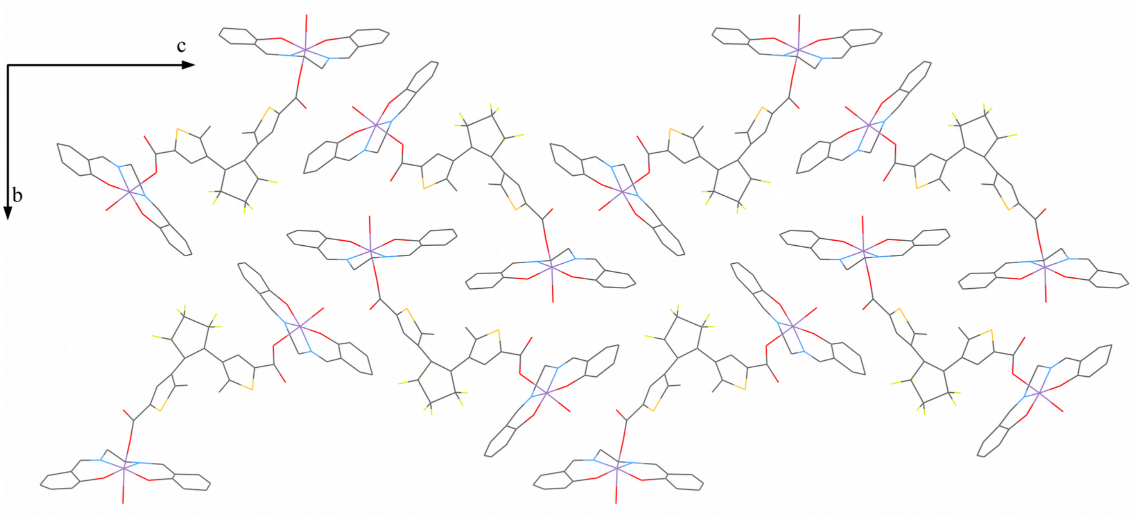

Figure S4. Packing diagrams of **1c-Vis** projected along bc plane. The hydrogen atoms and are omitted for clarity.

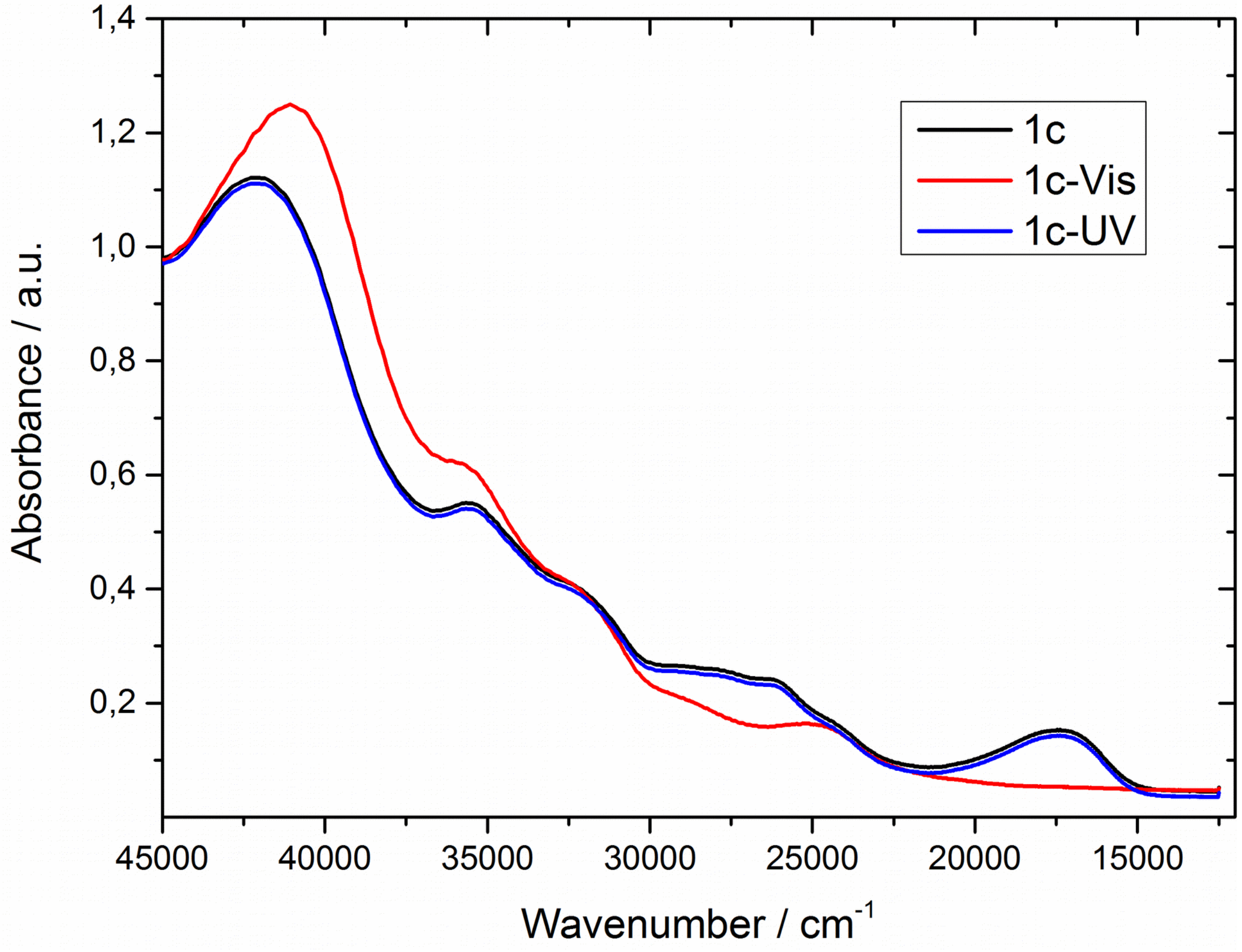

Figure S5. UV-vis absorption spectra in solid state of **1c**, **1c-Vis**, and **1c-UV** supported on KBr matrix.

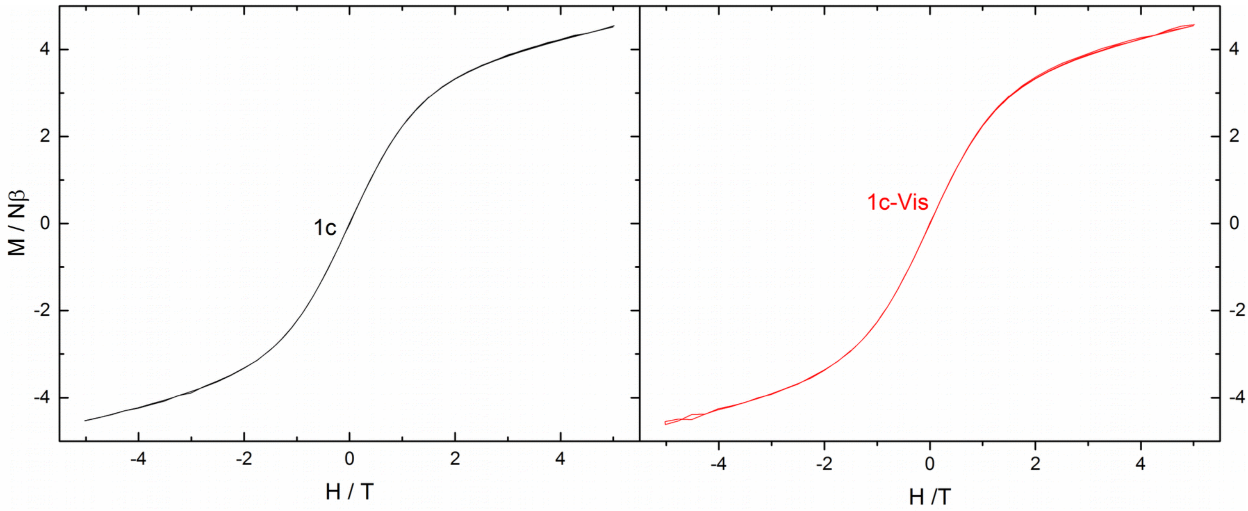

Figure S6. Plots of *M* vs. *H* at 1.82 K for **1c** and **1c-Vis.**

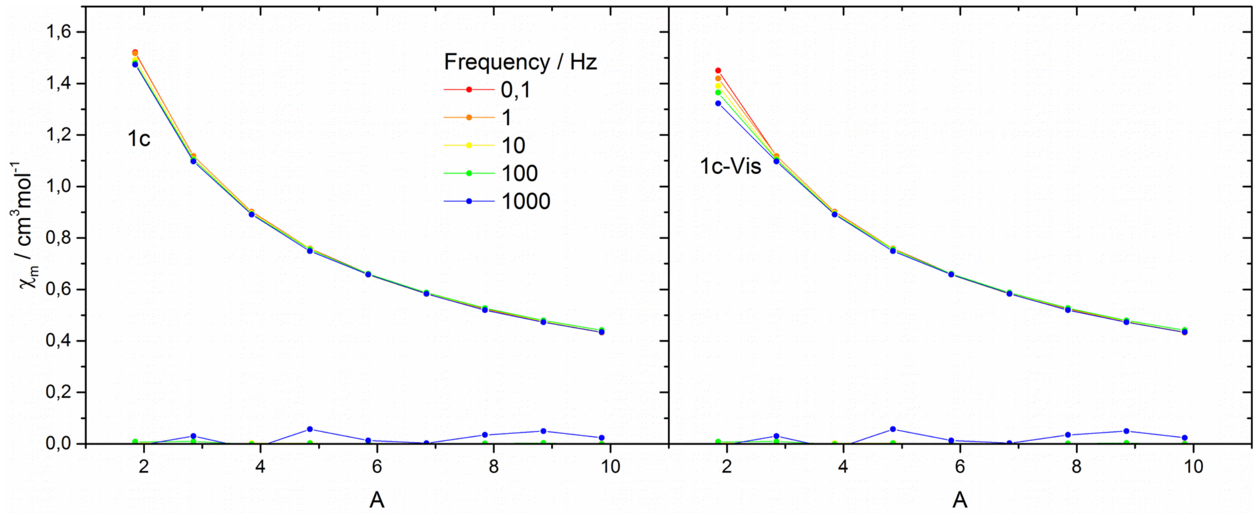

Figure S7. Frequency dependence of in-phase and out-of-phase susceptibility for **1c** and **1c-Vis**.

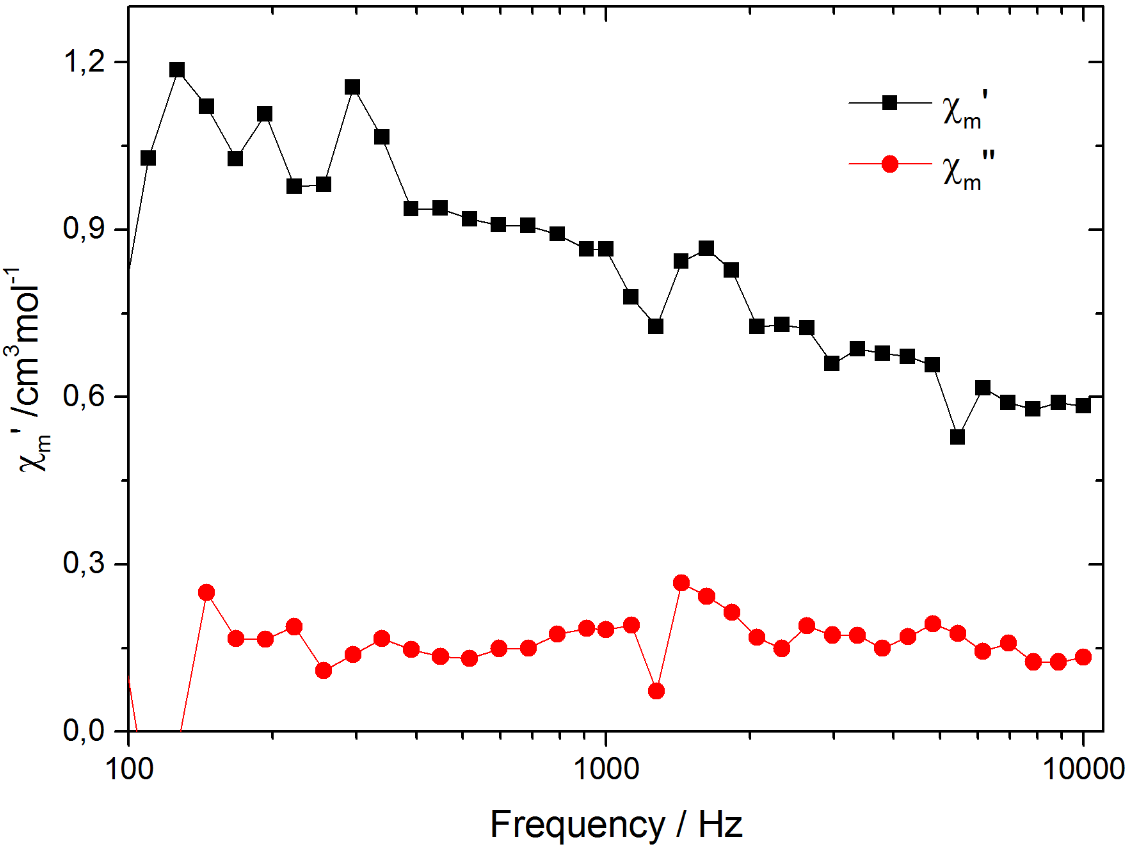

Figure S8. Frequency dependence of in-phase and out-of-phase susceptibility for **1c** at 1.9 K and 0.4 T.

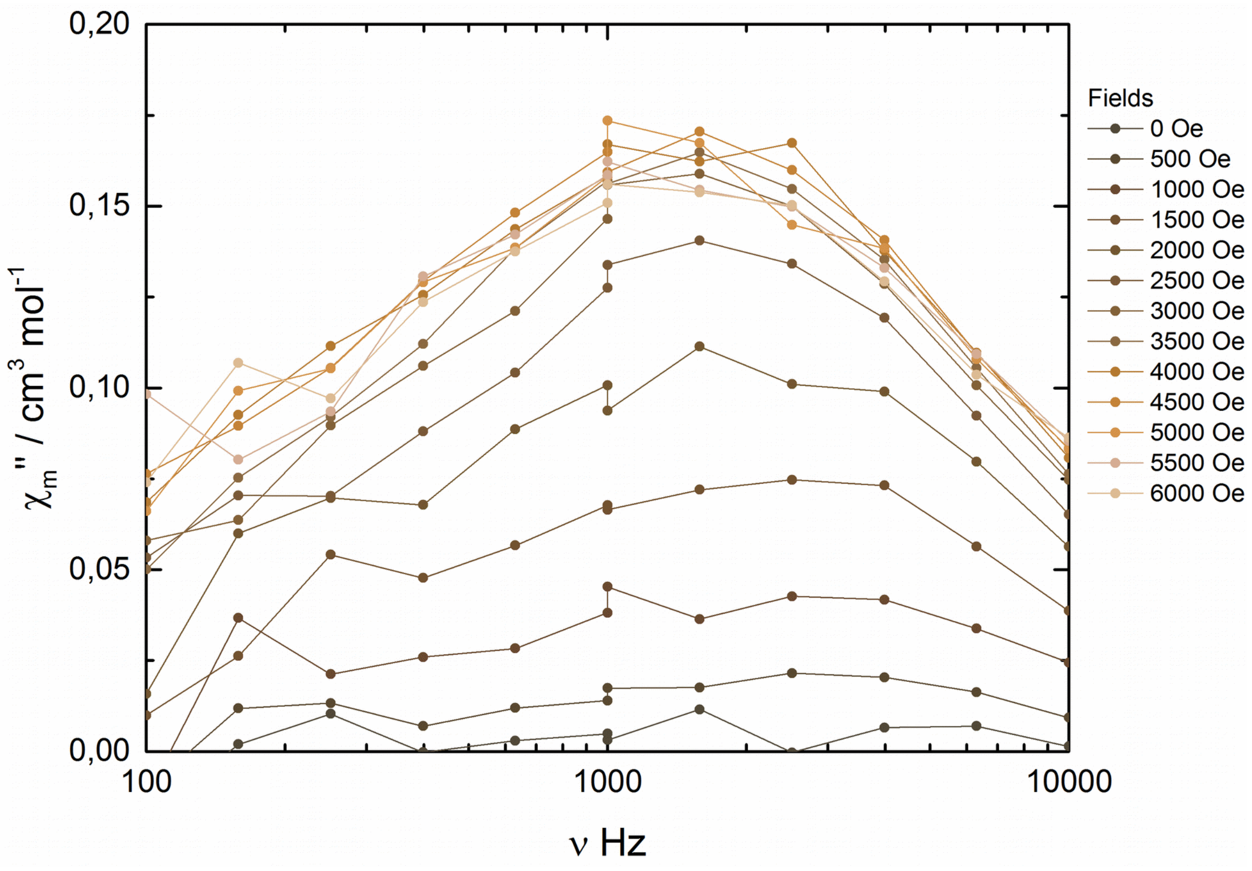

Figure S9. Frequency dependence of out-of-phase susceptibility for **1c-Vis** at 1.9 K.
